# Microfibrous Carbon Paper Decorated with High-Density Manganese Dioxide Nanorods: An Electrochemical Nonenzymatic Platform of Glucose Sensing

**DOI:** 10.3390/s24185864

**Published:** 2024-09-10

**Authors:** Khawtar Hasan Ahmed, Mohamed Mohamedi

**Affiliations:** Institut National de la Recherche Scientifique (INRS), Énergie Matériaux Télécommunications (EMT), 1650, Boulevard Lionel-Boulet, Varennes, QC J3X 1P7, Canada

**Keywords:** gold, manganese oxide nanorods, hydrothermal, non-enzymatic sensor, glucose detection

## Abstract

Nanorod structures exhibit a high surface-to-volume ratio, enhancing the accessibility of electrolyte ions to the electrode surface and providing an abundance of active sites for improved electrochemical sensing performance. In this study, tetragonal α-MnO_2_ with a large K^+^-embedded tunnel structure, directly grown on microfibrous carbon paper to form densely packed nanorod arrays, is investigated as an electrocatalytic material for non-enzymatic glucose sensing. The MnO_2_ nanorods electrode demonstrates outstanding catalytic activity for glucose oxidation, showcasing a high sensitivity of 143.82 µA cm^−2^ mM^−1^ within the linear range from 0.01 to 15 mM, with a limit of detection (LOD) of 0.282 mM specifically for glucose molecules. Importantly, the MnO_2_ nanorods electrode exhibits excellent selectivity towards glucose over ascorbic acid and uric acid, which is crucial for accurate glucose detection in complex samples. For comparison, a gold electrode shows a lower sensitivity of 52.48 µA cm^−2^ mM^−1^ within a linear range from 1 to 10 mM. These findings underscore the superior performance of the MnO_2_ nanorods electrode in both sensitivity and selectivity, offering significant potential for advancing electrochemical sensors and bioanalytical techniques for glucose monitoring in physiological and clinical settings.

## 1. Introduction

Glucose serves as a vital energy source in our bodies; however, deviations from the average level can lead to health issues. Low levels of glucose, known as hypoglycemia (below 4 mmol/L), or high levels, termed hyperglycemia (above 7 mmol/L), can both pose significant health risks [1]. Diabetes is a metabolic disorder marked by the body’s failure to produce insulin (type I diabetes or insulin-dependent diabetes) or effectively utilize insulin (type II/insulin-resistant). Consequently, glucose is unable to enter cells, leading to its accumulation in the bloodstream and consequent damage to blood vessels. Accurate determination and monitoring of blood glucose concentration is crucial for diagnosing and managing the disease. Failure to do so may result in severe complications, including cardiovascular disease, heart attack, arthritis, stroke, vision loss, kidney failure, nerve damage, and gangrene-related amputations [2]. Given the serious consequences of uncontrolled blood glucose levels, the development of reliable and cost-effective glucose measurement devices poses a significant challenge.

Electrochemical glucose sensors encompass a variety of types based on their operational principles and detection techniques [3]. These include: (i) enzymatic glucose sensors (EGS) [4], (ii) non-enzymatic glucose sensors (NEGS) [5], (iii) amperometric glucose sensors (AGS) [6], (iv) potentiometric glucose sensors (PGS) [7], (v) impedimetric glucose sensors (IGS) [8] and (vi) field-effect transistor (FET) glucose sensors [9]. EGS and NEGS have been extensively studied and developed for diverse applications such as blood glucose monitoring for diabetes management, food quality control, and biomedical research. EGS utilizes the catalytic activity of glucose oxidase (GO_x_) or glucose dehydrogenase (GDH) enzymes to selectively oxidize glucose, generating a current or potential change proportional to the glucose concentration. While EGS offers high selectivity and sensitivity to glucose, they may encounter limitations such as enzyme instability, response time, temperature sensitivity, complexity, cost, biocompatibility concerns if they are intended for implantable use, and susceptibility to interference from substances other than glucose present in the sample. Compounds such as ascorbic acid (0.02–0.08 mM), uric acid (0.18–0.42 mM), and certain medications can interfere with the enzymatic reaction, leading to inaccurate glucose measurements. On the other hand, NEGS do not rely on biological catalysts for glucose detection. Instead, they employ noble metal or metal oxide-based materials with intrinsic catalytic activity towards glucose oxidation. These catalysts enable the direct electrooxidation of glucose molecules on the sensor surface, producing an electrochemical signal correlating with the glucose concentration. NGES offer advantages including simplicity, stability, and freedom from enzyme-related issues, although they may exhibit lower selectivity compared to EGS. The choice between EGS and NGES types depends on factors such as selectivity needs, sensitivity requirements, cost considerations, and specific application contexts.

Currently, research is progressing towards the advancement of fourth-generation electrochemical sensor technologies. These innovations are designed to address the constraints of earlier generations while enhancing performance, accuracy, and user satisfaction. These sensors typically integrate innovative features and novel materials to enhance glucose monitoring capabilities. Fourth-generation glucose sensors often incorporate advanced features such as enhanced selectivity, continuous monitoring capabilities, miniaturization, wearability, wireless connectivity, improved biocompatibility, longevity, and user-friendly interfaces [10,11]. While EGS have been widely utilized and remain foundational for glucose monitoring, they are classified within earlier generations of glucose sensing technology. Consequently, they are typically not considered part of the fourth generation of glucose sensors. Emerging technologies in this domain may encompass NEGS, implantable sensors, microfluidic sensors, and other innovative approaches that expand the horizons of glucose sensing capabilities.

The most studied materials for electrochemical detection of glucose in NEGS include carbon-based materials (carbon nanotubes, graphene, and graphene oxide) [12], metal nanoparticles (Au, Ag, and Pt) [13], conducting polymers [14], and metal oxides [15]. These materials are often used in various combinations or composite forms to enhance the performance of NEGSs in terms of sensitivity, selectivity, and stability. For additional information, readers are referred to the comprehensive review by Rajesh Kumar Mani et al., which delves into recent advancements in NEGS [16]. This overview covers a range of topics, including various materials utilized as sensing elements, fabrication methods, and performance characteristics, and offers insights into future directions within this field.

Among the interesting materials, manganese dioxide (MnO_2_) holds promise for the development of fourth-generation glucose electrochemical sensors due to several advantageous properties. Some key reasons for its popularity in this field include (i) high catalytic activity towards glucose oxidation, which is crucial for achieving rapid and sensitive detection; (ii) biocompatibility, making it suitable for interfacing with biological samples without causing adverse effects; (iii) abundant availability and cost-effective, contributing to the feasibility of large-scale sensor fabrication and (vi) stability under various environmental conditions, ensuring the longevity and reliability of glucose sensors. Another benefit of MnO_2_ lies in its versatility. It can be synthesized in diverse forms providing flexibility in sensor design and manufacturing. Despite these benefits, there are comparatively few reports in the literature on MnO_2_-based glucose sensors [17,18,19,20].

Researchers frequently investigate various morphologies and nanostructures of materials, such as nanowires, nanosheets, and nanoparticles, aiming to bolster their electrochemical capabilities for glucose detection. In contrast to film structures, nanorods boast a high surface-to-volume ratio, which offers a greater abundance of active sites for electrochemical reactions, consequently leading to enhanced electrochemical surface area (*ECSA*). Their three-dimensional architecture enables improved accessibility of electrolyte ions to the electrode surface, facilitating swifter charge transfer kinetics and expanding the effective surface area accessible for electrochemical processes. Moreover, the hierarchical arrangement of nanorods promotes efficient charge transport within the electrode material, mitigating diffusion limitations and augmenting the utilization of active material, thereby elevating *ECSA*. Lastly, nanorods often manifest heightened electrochemical activity attributable to their unique surface properties and crystallographic facets, yielding a denser distribution of active sites available for electrochemical reactions [21,22,23].

As far as we know, there is a lack of studies specifically investigating the use of MnO_2_ nanorods for glucose electrochemical sensing. Therefore, the primary aim of this work is to assess the electrochemical sensing capabilities of MnO_2_ nanorods synthesized through a one-step hydrothermal technique for glucose (Glu) detection. Concurrently, we aim to evaluate their electrochemical activity against interfering molecules such as uric acid (UA) and ascorbic acid (AA). Another distinctive aspect of our research is the self-supporting nature of our electrodes. This signifies that the catalyst was directly grown on the substrate (carbon paper) without the inclusion of a binder or other additives, potentially mitigating any interference with the electrochemical results.

## 2. Experimental Section

### 2.1. Reagents

NaOH (Fisher Scientific, Nepean, ON, Canada, purity +97%), glucose (Sigma–Aldrich, Oakville, ON, Canada), ascorbic acid (Sigma, Ontario, Canada, purity 99%) and uric acid Sigma, purity 99%), HCl (Sigma–Aldrich, concentration 37%), and KMnO_4_ (Fisher Scientific, purity 99%) were used without further purification.

### 2.2. Material Synthesis

To promote the growth of MnO_2_, we utilized the hydrothermal method employing KMnO_4_ as the precursor. To begin with, 1.67 mmol of KMnO_4_ was dissolved in 18.75 mL of ultrapure deionized water (Millipore Milli-Q, resistivity 18.2 MΩ·cm, Sigma–Aldrich, Oakville, ON, Canada) and mixed for 15 min. After the KMnO_4_ had fully dissolved, 0.42 mL of concentrated HCl was added to the solution and mixed continuously for 2 min. Next, a piece of carbon paper (Toray carbon paper, TGP-H-60) was carefully placed into a 25 mL Teflon-lined stainless-steel autoclave, and the KMnO_4_-HCl solution was then carefully added. The autoclave was sealed and then heated in an oven at 140 °C for 12 h. Once the synthesis was complete, the reactor was allowed to cool to room temperature. The resulting CP/MnO_2_ samples were then washed thoroughly with deionized water and subsequently annealed in air at 400 °C for 2 h.

### 2.3. Materials Analysis

The surface characteristics of the synthesized samples underwent examination via SEM (TESCAN VEGA3, Brno, Czech Republic) operating at 20.0 kV. Crystallinity was assessed using X-ray diffraction (XRD) with a Bruker D8 Advance diffractometer, which was equipped with a Cu Kα source and operated at 40 kV and 40 mA. Diffraction patterns were collected with a step size of 0.04° and an acquisition time of 2 s per step. Furthermore, Micro-Raman spectroscopy was used to assess the structural quality of the samples. Raman spectra in the range from 100 to 2000 cm^−1^ were obtained using a Renishaw (inVia Reflex, Mississauga, ON, Canada) instrument, with a 532 nm laser operating at a low power of 0.1 mW (1% of 10 mW). The spot size analyzed was 2 μm, and three spectra were collected for each sample, each with a 50-second acquisition time, to obtain an average spectrum.

### 2.4. Electrochemical Measurements

To investigate the electrochemical performance, an electrochemical analyzer (Eco Chemie PGSTAT302 potentiostat/galvanostat, Metrohm Autolab, Utrecht, The Netherlands) was employed. Measurements were conducted at room temperature using a three-compartment electrochemical cell, which included a platinum coil as the counter electrode, a Ag/AgCl reference electrode with a 4 M KCl solution, and a rectangular CP/MnO_2_ working electrode. To reduce the effect of ohmic drop, the reference electrode was placed near the working electrode and isolated from the electrolyte by a Luggin capillary. A 0.1 M aqueous NaOH solution was used as the electrolyte, which was deoxygenated by bubbling argon from 20 to 30 min before each electrochemical measurement.

### 2.5. Capacitive Properties and ECSA Measurements

Cyclic voltammetry (CV) measurements were recorded at different scan rates from 2 to 500 mVs^−1^. Specific capacitance based on the CV curves was calculated using *C_p_* = *Q*/(2 *m* × *v* × Δ*V*), where *Q* (A V) is the voltametric charge determined by integrating either the oxidation and the reduction areas of the CV curve, *m* (g) is the mass loading of the active material (MnO_x_) in the working electrode, *v* is the scan rate (V s^−1^), and Δ*V* (V) represents the potential window of the cyclic voltammogram. The factor of 2 in the denominator accounts for the fact that the capacitance measured from a CV curve reflects both the forward and reverse scans. This effectively doubles the charge contribution. Since the integration of the CV curve includes both charging and discharging phases, and it is assumed that these contribute equally to the total charge, we only consider the discharging part when calculating the specific capacitance. Therefore, the area must be divided by 2 to accurately reflect the specific capacitance. The electrochemical double-layer capacitance, *C_dl_* was derived from the equation *i_c_* = *v* × *C_dl_*, where *i_c_* represents the double-layer charging current. Consequently, plotting *i_c_* as a function of *v* results in a linear relationship, with the slope equal to *C_dl_*. The *ECSA* of a catalyst sample is determined using the double-layer capacitance, which is expressed as *ECSA* = *C_dl_*/*C_s_*. Here, *C_s_* denotes the standard specific electrochemical double-layer capacitance of the sample or the capacitance of an atomically smooth planar surface of the material per unit area under the same electrolyte conditions. Ideally, creating smooth, planar surfaces for each catalyst would facilitate accurate measurement of *C_s_* and estimation of the electrochemical surface area (*ECSA*). However, this method is often impractical for most synthesized systems. Nevertheless, it is worth noting that typical specific capacitances of 0.040 mF cm^−2^ have been documented for metal electrodes in 1 M NaOH solution [24]. Regarding carbon, *C_s_* is approximately 13 µF cm^−2^, calculated using an average value of 5–20 µF cm^−2^ reported [25,26]. The roughness factor (*RF*) is calculated by dividing the estimated *ECSA* by the geometric area of the electrode, which is 0.180 cm^2^.

### 2.6. Electrochemical Detection of Glucose, AA, and UA

The electrochemical properties of the CP/MnO_x_ towards Glu, AA, and UA electrooxidation were explored using the CV technique. Before each electrochemical experiment, a 0.1 M NaOH solution is purged with Argon gas for approximately 20 min to eliminate any dissolved oxygen. Subsequently, CV measurements are performed by immersing the CP/MnO_x_ samples into the deaerated NaOH solution, initially without Glu, AA, or UA present (to establish the background response), and then in the presence of Glu, AA, or UA. All these measurements were conducted using various concentrations of Glu, AA, and UA. The sensitivity of the sensor was determined by analyzing the current peak density as a function of glucose concentration. A linear regression analysis was performed on the plot of current peak density versus glucose concentration. The sensitivity is derived from the slope of this linear regression line, representing the change in current peak density per unit change in glucose concentration. The limit of detection (LOD) was calculated using the formula: LOD = 3.3 (*σ*/*s*), where *σ* is the standard deviation of the baseline signal, and *s* is the slope of the linear regression line. This formula provides an estimate of the lowest concentration of glucose that can be reliably detected by the sensor, where factor 3.3 accounts for the desired confidence level of detection.

## 3. Results and Discussion

### 3.1. Materials Characterization

**Figure 1:**
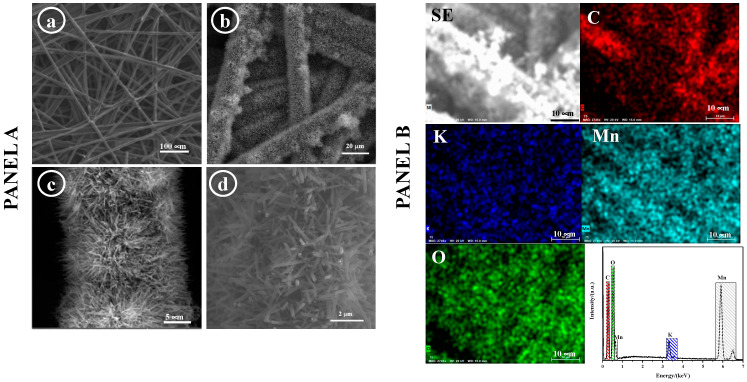
(**Panel A**) SEM images of (**a**) bare CP substrate, (**b**–**d**) of CP/MnO_x_ at increasing magnifications. (**Panel B**) Element mapping and EDS spectrum.

The surface morphology of the bare carbon paper substrate and the prepared MnO_x_ are depicted in Figure 1a–d, respectively (Panel A). It is evident that the CP substrate is coated with a dense array of nanorod (NR) arrays, with an average diameter of approximately 200 nm. Figure 1 (Panel B) demonstrates the uniform distribution of MnO_x_ across the CP substrate, as confirmed using EDS mapping. Both elemental mapping and the EDS spectrum reveal the consistent presence of O, Mn, and K elements on the CP surface.

**Figure 2:**
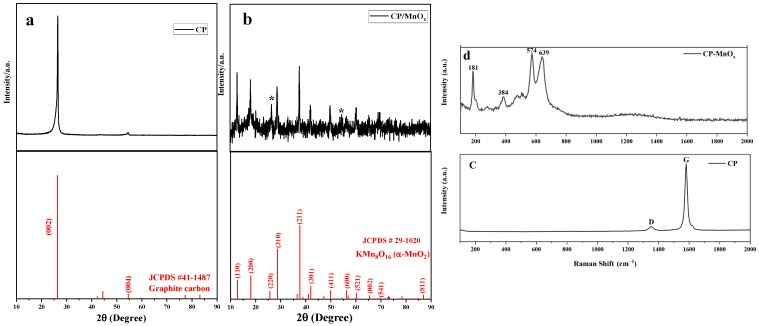
XRD patterns of (**a**) CP substrate and (**b**) CP/MnO_x_. Raman spectra of (**c**) CP substrate and (**d**) CP/MnO_x_.

XRD analysis was employed to examine the crystal structures of both the CP substrate and MnO_x_. In Figure 2a, the CP substrate displays a prominent peak at 26.4° (2θ) and two smaller peaks at 44.4° and 54.5°, corresponding to the (002), (101), and (004) planes of the graphite carbon substrates, respectively (JCPDS #41-1487). Conversely, the XRD profile of MnO_x_ reveals peaks, which are well-matched with the planes of cryptomelane KMn_8_O_16_ (JCPDS #29-1020). This compound represents a typical tetragonal α-MnO_2_ phase within the manganese oxide family, where K^+^ ions are situated within its 2 × 2 tunnels to stabilize the α phase’s crystalline structure and maintain charge balance. Figure 2c,d depicts the Raman spectra of both CP and CP/MnO_x_, respectively. The Raman spectrum of the CP substrate reveals two distinct bands at approximately 1350 and 1580 cm^−1^. These bands are recognized as the D band, which corresponds to *A*_1g_ symmetry from edge or defect sites of carbon, and the G band, which is associated with *E*_2g_ symmetry for sp^2^ carbon [27]. The *I_G_*/*I_D_* ratio of pristine CP is determined to be 8.85 based on the deconvolution area. In the case of samples containing MnO_x_, four distinct Raman peaks are observed at approximately 181, 384, 574, and 639.5 cm^−1^, which align with documented peaks of α-MnO_2_ [28]. The Raman band centered at 639.5 cm⁻^1^ is assigned to the symmetric stretching vibration of the Mn−O bond perpendicular to the direction of the [MnO_6_] octahedral double chains, belonging to the *A*_g_ symmetry spectroscopic mode based on factor group analysis. Similarly, the band at approximately 574 cm^−1^, also an *A*_g_ symmetric mode, corresponds to the stretching vibration of the Mn−O bond along the direction of [MnO_6_] octahedra double chains. The peak at around 384 cm^−1^, an *E*_g_ symmetry vibration mode, is associated with the bending vibration of the Mn−O bond. Additionally, the sharp peak at roughly 181 cm^−1^, also an *E*_g_ symmetry vibration mode, indicates the translational motion of [MnO_6_] octahedra induced by tunnel ions of K^+^ [28,29]. These observations confirm that all of the bands are characteristic of tetragonal α-MnO_2_ [28,29,30]. Interestingly, the Raman spectra of CP/MnO_x_ do not show the D or G band features characteristic of the CP substrate. This lack of detection is due to the dense, thick layer of α-MnO_2_ that uniformly coats the CP substrate.

### 3.2. Electrochemical Performance

#### 3.2.1. Electrochemical Surface Area

**Figure 3:**
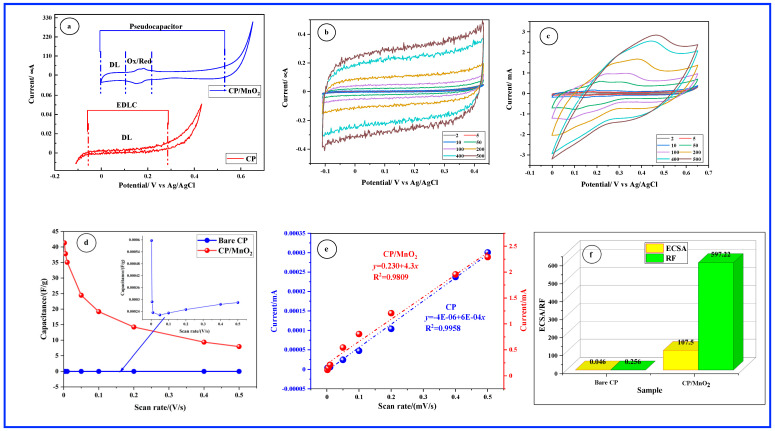
(**a**) Electrochemical windows of bare CP and CP/MnO_2_ in 0.1 M NaOH solution. (**b**) and (**c**) capacitive behavior of CP and CP/MnO_2_, respectively. Numbers indicate the scan rate in mV/s. (**d**) Specific capacitance. (**e**) Anodic currents extracted from (**b**,**c**) vs. scan rate. (**f**) *ECSA* and *RF* parameters.

Figure 3a illustrates a comparison of the electrochemical windows between bare CP and CP/MnO_2_ in a 0.1 M NaOH solution. Within the oxidation and reduction boundaries of NaOH, the CV of bare CP exhibits steady currents, indicating a pure double layer (DL) capacitance. On the contrary, CP/MnO_2_ CV exhibits symmetric peaks on both the anodic and cathodic sides within the 0.15 V and 0.2 V regions, alongside the DL region. The redox peaks observed at the MnO_2_ electrode typically indicate the involvement of surface Mn ions in redox reactions. This behavior underscores the pseudocapacitive nature of the charge storage mechanism, where the charge is stored through a reversible redox reaction at the electrode/electrolyte interface [31]. The capacitive properties of both CP and CP/MnO_2_ were further evaluated via CV in a 0.1 M NaOH solution, employing scan rates ranging from 2 to 500 mV s⁻^1^. These results are depicted in Figure 3b for CP and Figure 3c for CP/MnO_2_. The CV curves of the CP electrode exhibit nearly symmetrical rectangular shapes, characteristic of an electrochemical double-layer capacitor (EDLC), where all charges are stored on the material’s surface. Capacitive behavior is observed over a potential window of 0.5 V for the CP electrode. Specific capacitances, determined from the CVs shown in Figure 3b and plotted against the scan rate in Figure 3d, reveal that, for the CP electrode, the specific capacitance decreases as the scan rate increases, although it shows a slight recovery at higher scan rates. Even for bare carbon paper, which exhibits pure EDLC behavior, the specific capacitance decreases with an increasing scan rate due to reduced ion diffusion time and increased resistance. After reaching a certain scan rate, the capacitance stabilizes as the system transitions to a diffusion-controlled regime where ion transport becomes the primary limiting factor. This behavior is consistent with the general principles of electrochemical capacitors, even for materials exhibiting pure EDLC characteristics. It is worth mentioning that the specific capacitance provided by the bare CP is relatively small, with the highest value reaching close to 0.0006 F g⁻^1^ at 2 mV s⁻^1^. Conversely, it is observed that the capacitive potential window is expanded to 0.7 V with CP/MnO_2_ (Figure 3c) compared to 0.5 V with CP. The rate performance of the CP/MnO_2_ electrode, illustrated in Figure 3d, shows how the specific capacitance varies with scan rate. It is evident that the specific capacitance diminishes as the scan rate increases. A notable feature of Figure 3d is the high specific capacitance of CP/MnO_2_, reaching 42 F g⁻^1^ at 2 mV s⁻^1^. The large specific capacitance of the CP/MnO_2_ composite can be attributed to both structural and electrochemical factors. The unique structure of the MnO_2_ nanorod arrays, which feature highly exposed active surfaces, facilitates rapid electrolyte diffusion and fast Na^+^ transfer, contributing significantly to the capacitance. Additionally, the high specific capacitance is also a result of the faradaic charge storage capability of MnO_2_. This faradaic behavior involves redox reactions that provide an enhanced charge storage mechanism beyond the contributions from surface area alone. Consequently, the high specific capacitance of the CP/MnO_2_ composite is due to the synergistic effects of the nanostructured surface of MnO₂ and its faradaic charge storage properties. However, the specific capacitance markedly declines at scan rates above 10 mV s⁻^1^. This behavior, characteristic of transition metal oxides (TMOs), is due to the decreased diffusion of electrolyte ions into the active material matrix at higher scan rates. The *C_dl_* was evaluated for CP and CP/MnO_2_ at 0.6 V within the non-faradic region. Figure 3e illustrates the current values plotted against the scan rate. Notably, all curves exhibit a linear trend across the entire range of measured scan rates. By analyzing the scan rates versus the current plot, we can derive the *C_dl_* values for the samples, which are equivalent to the slopes of the graph. The determined *C_dl_* values are 0.0006 mF for bare CP and 4.3 mF for CP/MnO_2_, respectively. Subsequently, Figure 3f displays the calculated *ECSA* and *RF* values for each sample. Notably, the *ECSA* of carbon paper is observed to be 0.046 cm^2^. This relatively small value can be attributed to the smooth and planar surface of the CP substrate, which may limit the available active sites for electrochemical reactions, thus resulting in a reduced *ECSA*. Additionally, the CP substrate may exhibit a lower density of active sites per unit area, further contributing to the smaller *ECSA*. Conversely, MnO_2_ nanorods exhibited an outstandingly large *ECSA* of 107.5 cm^2^, accompanied by a remarkably high *RF* of 597.22. These remarkable *ECSA* and *RF* values can be attributed to several factors. Firstly, MnO_2_ nanorods have a high aspect ratio, providing a large surface area compared to their volume. This characteristic enhances the availability of active sites for electrochemical reactions. Secondly, their 3D morphology facilitates improved accessibility of electrolyte ions to the electrode surface, promoting faster charge transfer kinetics and effectively increasing the available surface area for electrochemical processes. Additionally, the hierarchical arrangement of MnO_2_ nanorods, with smaller branches or facets contributing to the overall surface area, further enhances the utilization of active material and facilitates efficient charge transport within the electrode material. Overall, the combination of these factors leads to the high *ECSA* and *RF* observed in MnO_2_ nanorods, which can have significant implications for their properties and applications in electrochemical sensing.

#### 3.2.2. Electrochemical Detection of Glu, AA, and UA at Au-Wire Electrode

**Figure 4:**
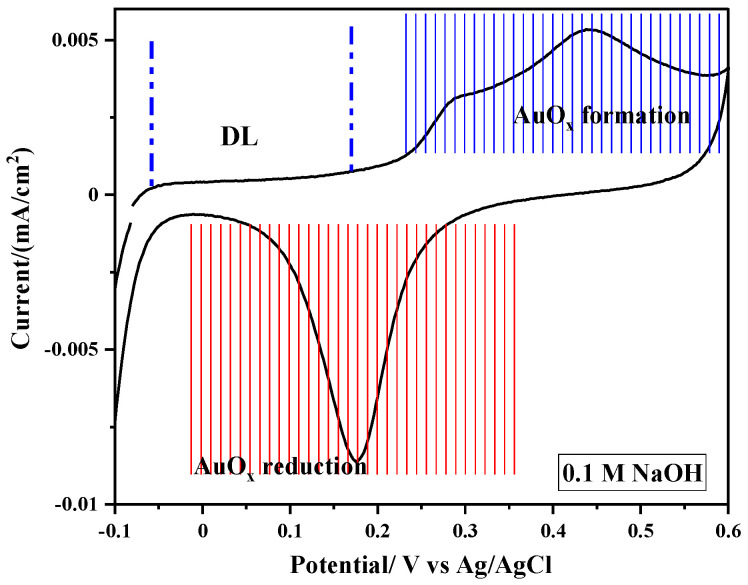
An electrochemical window of an Au-wire electrode in 0.1 M NaOH solution recorded with a scan rate of 2 mV/s.

In the subsequent discussion, we initially examine gold as a benchmark catalyst whose electrochemical characteristics towards the oxidation of Glu, AA, and UA are well documented. The outcomes of this study provide a foundational framework for interpreting the results obtained later on CP and CP/MnO_2_ electrodes. Figure 4 illustrates a CV of a Au-wire electrode in a 0.1 M NaOH solution. In the positive-going scan, a typical double-layer region of Au is observed between −0.1 and 0.2 V (Ag/AgCl), followed by a distinct peak at 0.440 V and a reduction peak at 0.175 V. These peaks are attributed to the anodic formation and cathodic removal of surface gold oxide, consistent with findings in the literature [32,33]. Additionally, a minor wave appearing around 0.288 V prior to the surface oxide formation process suggests a possible premonolayer oxidation of the Au surface.

**Figure 5:**
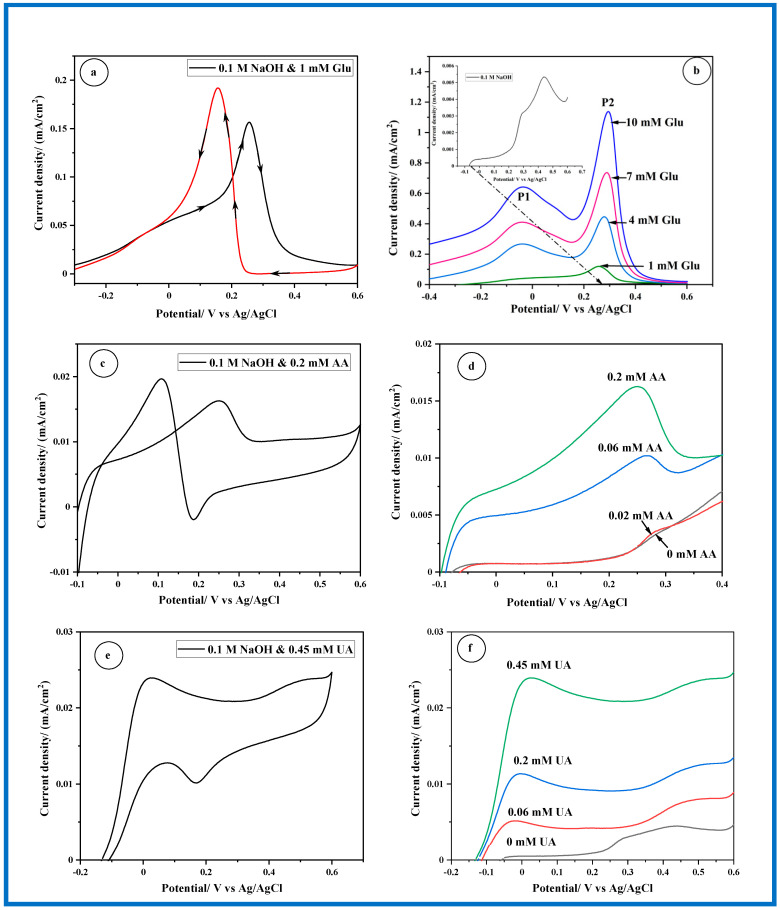
Electrochemical oxidation of Glu (**a**,**b**), AA (**c**,**d**), and UA (**e**,**f**) on Au-wire electrode in 0.1 M NaOH solution recorded with a scan rate of 2 mV/s.

When a modest concentration of 1 mM of Glu is introduced into a NaOH solution at the same wire electrode, two new features emerge during the forward scan (Figure 5a). Firstly, a wave appears at −0.036 V_Ag/AgCl_, followed by a peak at 0.255 V_Ag/AgCl_. Both the current densities associated with these features increase with the increasing concentration of Glu, as illustrated in Figure 5b. It is noteworthy that the wave at −0.036 V transforms into a distinct peak with the rise in Glu concentration. The observed CV response aligns with the typical voltammetric pattern associated with glucose oxidation at Au electrode [34,35]. In earlier studies, the first peak was associated with the oxidation of glucose to gluconolactone, a process involving two electrons. The second peak, on the other hand, appears to be related to the oxidation of gluconolactone to gluconic acid, which involves four electrons [34,35].

The CV response observed for the oxidation of AA at a gold electrode in a NaOH solution displays clear characteristics: an anodic peak emerges during the forward potential scan, positioned at 0.250 V_Ag/AgCl_, signifying the oxidation of AA to dehydroascorbic acid (Figure 5c). Conversely, a cathodic peak appears during the reverse potential scan at 0.187 V_Ag/AgCl_, denoting the reduction of dehydroascorbic acid back to AA at the electrode surface. Notably, the current density of the forward peak increases with increasing concentrations of AA (Figure 5d). Figure 5e illustrates the CV results obtained from oxidizing 0.45 mM UA at the Au-wire electrode within a 0.1 M NaOH solution, spanning the potential range from −0.15 V to 0.6 V. The CV reveals several features: an anodic peak emerging at 0.027 V, succeeded by a broad wave spanning from 0.4 V to 0.6 V, and a peak positioned at 0.170 V during the reversal scan. However, interpreting the latter two processes proves challenging due to their overlap with the regions associated with Au oxide formation and reduction. Initially, the anodic peak at 0.027 V was attributed to the saturated adsorption of UA at the Au electrode in the 0.1 M NaOH solution, as suggested by Md. Rezwan Miah [36]. Nevertheless, our findings diverge, revealing that this peak intensifies with increasing UA concentration, implying its association with a faradic process (Figure 5f). The electrochemical oxidation UA at a Au electrode in a NaOH solution is a complex process with multiple steps. There may not be a single universally accepted mechanism, as researchers are continuously investigating and refining our understanding of these processes through theoretical modeling, spectroscopic studies, and electrochemical techniques. However, there are general trends commonly proposed in the literature. These steps typically involve the adsorption of UA molecules onto the surface of the Au electrode followed by their electrochemical oxidation, leading to the formation of UA radicals. These radicals may then undergo further oxidation, resulting in the formation of intermediate products such as allantoin or UA derivatives [37]. However, it’s worth noting that discussing the detailed mechanism is not the primary focus of the present work. Instead, our interest lies in the detection of uric acid using technical voltammetry.

**Figure 6:**
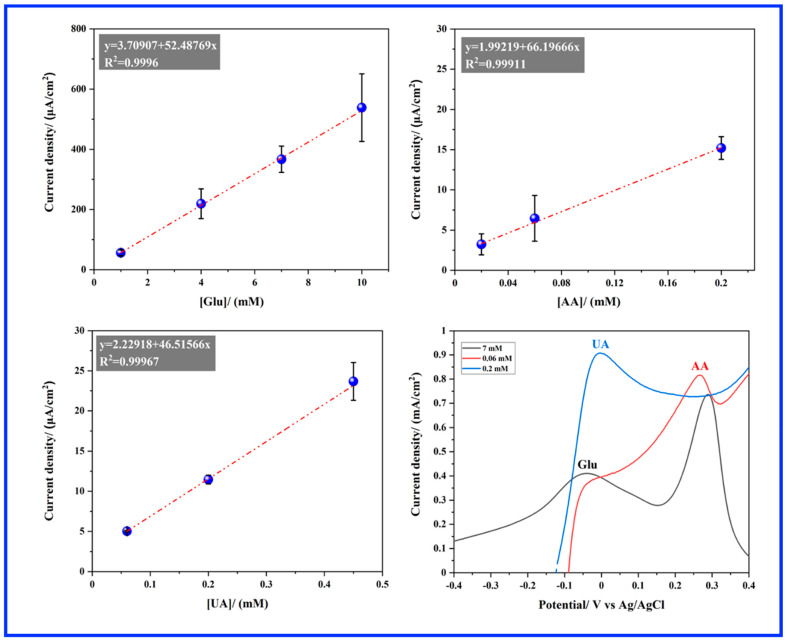
Comparison of current peak densities with respect to the concentration of Glu, AA, and, UA. Overview of the potential ranges for the oxidation of Glu, AA, and UA Figure 6 (bottom right). Concentrations used: [Glu] = 7 mM, [AA] = 0.06 mM, [UA] = 0.2 mM. Scale: peak current density is normalized, with Glu as the reference (×1), and AA and UA are scaled by a factor of 80.

In summary, Figure 6 illustrates the correlation between the current peak density extracted from Figure 5b,d,f and the concentration of Glu (P1), AA, and UA respectively, indicating a good linear relationship within the concentration range examined in this study. Concerning Glu detection, the Au-wire electrode demonstrates a sensitivity of 52.487 µA cm^−2^ mM^−1^ and a low detection limit (LOD) of 0.095 mM. Figure 6 further provides a summary of the potentials at which Glu, AA, and UA are detected. Notably, AA exhibits oxidation at more positive potentials, ensuring minimal interference with Glu detection on the Au electrode. However, UA detection closely aligns with Glu potentials, potentially affecting the accurate determination of Glu concentration.

#### 3.2.3. Electrochemical Detection of Glu at MnO_2_ Nanorods Electrode

**Figure 7:**
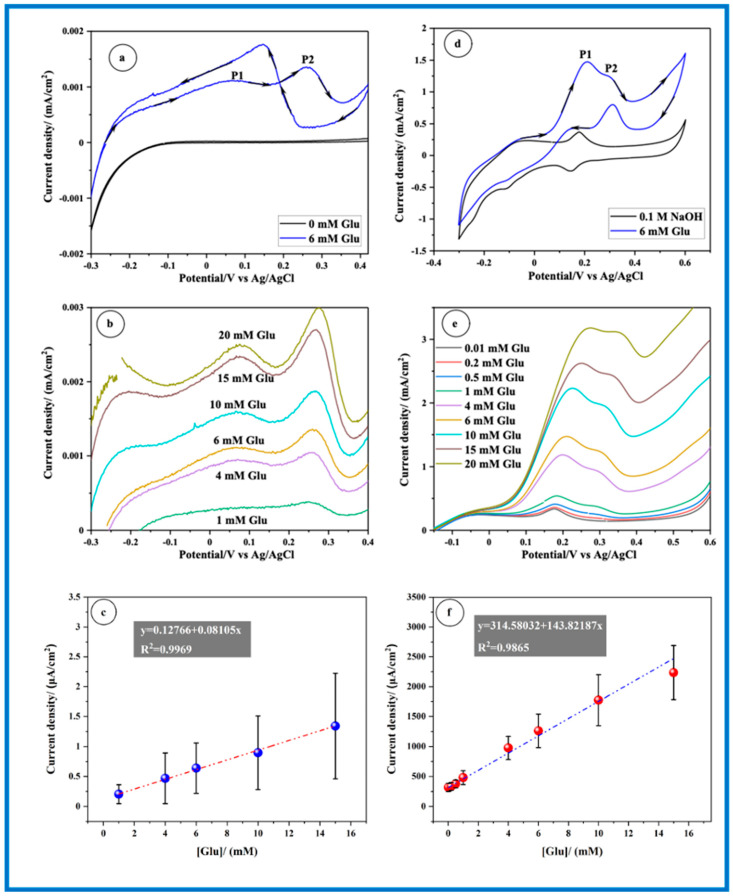
Electrochemical oxidation of Glu (**a**–**c**) on CP electrode, and (**d**–**f**) CP/MnO_2_ nanorods electrode in 0.1 M NaOH solution recorded with a scan rate of 2 mV s^−1^.

In Figure 7a, the CV profile of the CP electrode is presented, both with and without the presence of Glu. Upon the addition of glucose, a noticeable activity towards Glu oxidation is observed at the CP electrode. Two anodic peaks, labeled as P1 and P2, are evident at potentials around 0.068 V and 0.264 V_Ag/AgCl_, respectively. Subsequently, to establish a quasi-steady state, slow linear sweep voltammetry (LSV) measurements were carried out with a scan rate of 2 mV s^−1^ in a 0.1 M NaOH solution containing various concentrations of Glu (as depicted in Figure 7b). It is observed that the current peak densities of both P1 and P2 increase with rising Glu concentration. Focusing on the maximum current peak, P1, for each Glu concentration, its dependency on Glu concentration is illustrated in Figure 7c. Notably, a linear relationship (R^2^ = 0.9969) is observed in the range from 1 to 15 mM, extending far beyond the physiological levels (3–8 mM). However, the CP electrode demonstrates a low sensitivity of 0.081 µA cm^−2^ mM^−1^ and an LOD of 0.425 mM. Figure 7d illustrates the CV profile of the MnO_2_ nanorods electrode, with and without the presence of Glu. Similar to the Au wire and CP electrodes, the CV of Glu oxidation at MnO_2_ nanorods exhibits two peaks, labeled as P1 and P2, in the forward scan, appearing at potentials of 0.208 V and 0.293 V_Ag/AgCl_, respectively. Both current densities of P1 and P2 increased with the rise in Glu concentration, as depicted in Figure 7e. A linear relationship (R^2^ = 0.9865) is evident in the range from 0.01 to 15 mM for peak P1. Furthermore, the electrode exhibits a very high sensitivity of 143.82 µA cm^−2^ mM^−1^ and an LOD of 0.282 mM.

**Figure 8:**
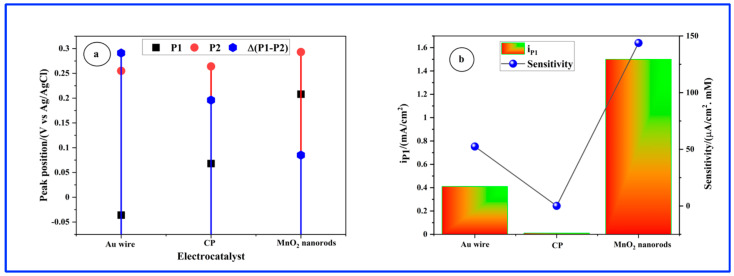
(**a**) Peak potentials: P1 and P2 and Δ(P2 − P1) separation. (**b**) Current density of peak P1 and sensitivity. [Glu] = 6 mM.

While certain aspects of CV behavior may exhibit similarities among gold, CP, and MnO_2_ nanorod electrodes, it is crucial to consider the distinctive characteristics of each electrode material when comparing their electrochemical responses. For instance, a significant disparity among the Au wire, CP, and MnO_2_ nanorods electrodes is the narrower separation ∆(P2 − P1) between the P1 and P2 peaks observed in the latter electrode (refer to Figure 8a). Regarding performance, the MnO_2_ nanorods electrocatalyst demonstrates markedly superior current peak density and sensitivity, approximately 2.7 times higher than that of the Au wire, particularly evident with a Glu concentration of 6 mM as illustrated in Figure 8b. This indicates that the MnO_2_ nanorod electrode exhibits exceptional catalytic activity for glucose oxidation. The electrochemical oxidation of glucose at a MnO_2_ electrode in NaOH electrolyte typically involves a multi-step process. Initially, glucose is oxidized to gluconolactone or gluconic acid, producing electrons that are transferred to the electrode surface. Prior research indicates that the catalytic glucose oxidation may involve the following steps [20,38,39].

MnO_2_ + 4OH^−^ → MnO_4_^2−^ + 2H_2_O + 2e^−^

MnO_4_^2−^ + glucose → gluconolactone + MnO_2_ + 2OH^−^

Gluconolactone + H_2_O → gluconic acid

#### 3.2.4. Electrochemical Oxidation of AA, UA, Binary, and Ternary Systems on MnO_2_ Nanorods

**Figure 9:**
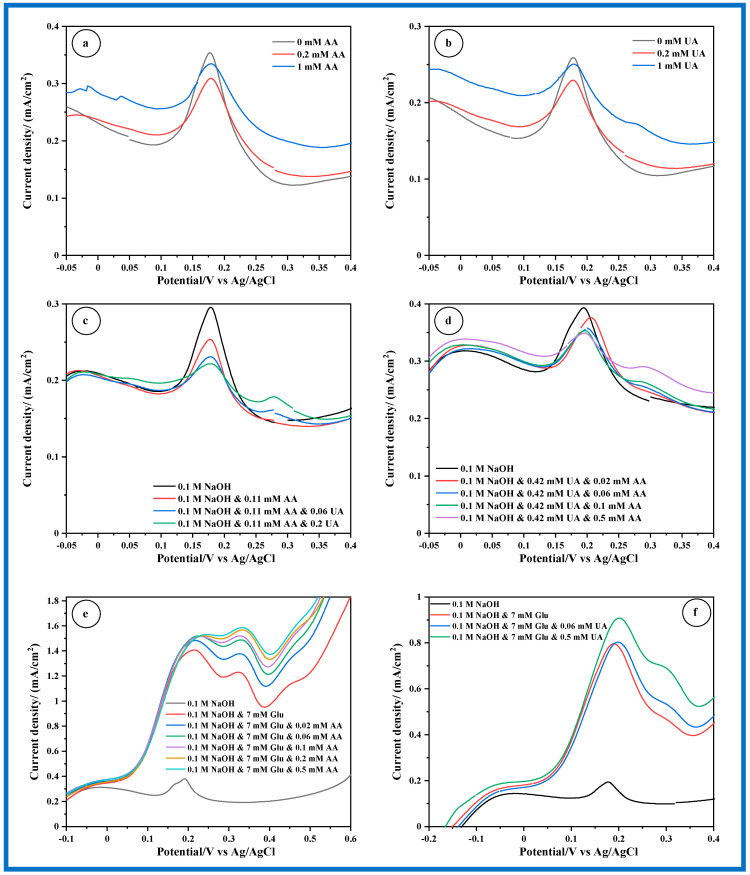
Electrochemical oxidation of (**a**) AA; (**b**) UA; (**c**,**d**) AA + UA; (**e**) Glu + AA and (**f**) Glu + UA on CP/MnO_2_ nanorods electrode in 0.1 M NaOH solution recorded with a scan rate of 2 mV s^−1^.

Figure 9a,b illustrates that following the addition of AA or UA to 0.1 M NaOH, no new peak emerges. Instead, the current density of the peak associated with the surface redox process at MnO_2_ diminishes with increasing AA or UA concentration. This response may stem from several potential factors. The adsorption of AA or UA or their reaction byproducts onto the MnO_2_ electrode surface could prompt the formation of a passivating layer, thereby impeding electron transfer processes and reducing current density. Additionally, the observed decrease in peak current density with increasing concentrations of AA or UA can be attributed to a combination of factors. While the initial explanation focused on the potential kinetic limitations of the redox reactions, where slower reaction rates of AA or UA compared to MnO_2_ could reduce the overall current density, surface-blocking effects are also significant. As the concentration of AA or UA increases, their surface coverage on the MnO_2_ electrode also rises, which can hinder the access of other reactants to the active sites and contribute to a decrease in current density. Therefore, both the kinetic effects of slower redox reactions and the surface blocking by higher concentrations of AA or UA play a role in the observed reduction in peak current density. A comprehensive understanding of the precise mechanism necessitates further experimentation and analysis, including the examination of reaction kinetics and the characterization of MnO_2_ electrode surface chemistry under varied conditions. In Figure 9c,d, one can observe the LSVs recorded in a NaOH solution comprising both AA and UA. In these plots, one concentration remains constant while the other varies. Interestingly, no additional peaks emerge when both AA and UA are concurrently present in the electrolyte, indicating no new chemical processes. Additionally, the peak associated with the surface redox process at the MnO_2_ electrode diminishes as the concentration of AA is either fixed or increased and conversely, as UA concentration varies. In contrast, a completely different trend is observed when Glu is in the presence of either AA or UA, as depicted in Figure 9e,f. At a fixed Glu concentration of 7 mM, representative of normal physiological levels, the current density of the initial peak associated with Glu oxidation to gluconolactone increases with rising AA or UA content. Specifically, with AA concentration rising from 0.02 to 0.06 mM, the current density of this peak rises, plateauing for higher AA concentrations (beyond physiological levels) (Figure 9e). Conversely, the second peak (P2), linked to the further oxidation of gluconolactone to gluconic acid, becomes more pronounced with increasing AA concentration. With 7 mM of Glu in the presence of UA, a notable surge in the current density of P1 is observed only when UA concentration reaches 0.5 mM, while the resolution of the P2 peak remains unaffected by the presence of UA (Figure 9f).

These observations suggest complex interactions between Glu, AA, and UA in the electrochemical milieu. The increase in the current density of the peaks associated with Glu oxidation with increasing concentrations of AA or UA indicates a potential catalytic effect of these compounds on the electrochemical oxidation of Glu. This catalytic effect may stem from the ability of AA and UA to facilitate electron transfer processes or modify the electrode surface, enhancing the electrochemical response of Glu. The plateauing of the current density beyond physiological levels of AA suggests a saturation effect, where further increases in AA concentration do not significantly influence the electrochemical behavior of Glu. This could imply that the catalytic activity of AA reaches a maximum at those concentrations. Overall, these observations provide valuable insights into the complex electrochemical behavior of Glu in the presence of AA and UA, which could have implications for the development of electrochemical sensors or bioanalytical techniques for glucose monitoring in physiological or clinical settings. Note that, in Figure 9e,f, the effect of adding AA and UA on the background current was evaluated, and it was found to remain unaffected across various conditions. This consistent background current ensures that the observed changes in peak currents are attributable to the electrochemical processes of the analytes rather than background interference.

**Figure 10:**
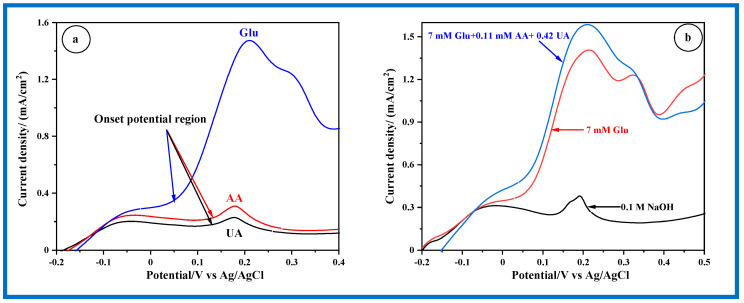
(**a**) Individual electrochemical oxidation of Glu, AA, and UA on CP/MnO_2_ nanorods electrode, (**b**) Electrochemical oxidation of ternary Glu + AA + UA on CP/MnO_2_ nanorods electrode in 0.1 M NaOH solution recorded with a scan rate of 2 mV/s.

Figure 10a outlines the distinct oxidation regions for Glu, AA, and UA. The LSV profiles clearly show that the onset potential region for Glu is significantly more negative compared to those for AA and UA. This highlights the faster kinetics of Glu oxidation compared to that of AA and UA. Subsequently, a mixed solution of Glu + AA + UA was tested to investigate the selectivity of the CP/MnO_2_ nanorods electrode. The normal physiological glucose concentration ranges from 4 to 7 mM, which is significantly higher than the maximum concentrations of interfering species such as AA (0.11 mM) and UA (0.42 mM). As depicted in Figure 10b, the introduction of 0.11 mM AA and 0.42 mM UA only results in a 7.1% increase in current compared to 7 mM glucose, indicating sufficient selectivity for the analysis of human blood.

## 4. Conclusions

A high-density array of MnO_2_ nanorods is directly grown on a microfibrous carbon paper substrate through a straightforward, low-temperature, and cost-effective hydrothermal method. Thanks to their structural characteristics and surface properties, which include a high surface area and a three-dimensional hierarchical arrangement, these MnO_2_ nanorods exhibit a high electrochemical surface area and a significant roughness factor. As a result, the MnO_2_ nanorod electrode demonstrates excellent catalytic activity for the oxidation of glucose molecules. It shows not only excellent selectivity towards glucose, AA, and UA but also a remarkable sensitivity of 143.82 µA cm^−2^ mM^−1^ within the linear range from 0.01 to 15 mM, with a limit of detection (LOD) of 0.282 mM specifically for glucose. In contrast, a gold electrode displays a lower sensitivity of 52.487 µA cm^−2^ mM^−1^ within a lower linear range from 1 to 10 mM, highlighting the superior performance of the MnO_2_ nanorods electrode. These findings could have significant implications for the advancement of electrochemical sensors or bioanalytical techniques for glucose monitoring in both physiological and clinical settings.

## Data Availability

No new data were created or analyzed in this study. Data sharing is not applicable to this article.
